# Supplementary material for: Bone Remodeling in Children with Acute Lymphoblastic Leukemia: A Two-Year Prospective Longitudinal Study
Source: Int J Mol Sci. 2025 May 1;26(9):4307. doi: 10.3390/ijms26094307 (PMC12072470; doi:10.3390/ijms26094307)
Supplement: Supplementary file 1 [file ijms-26-04307-s001.zip › ijms-3543325-supplementary.pdf]

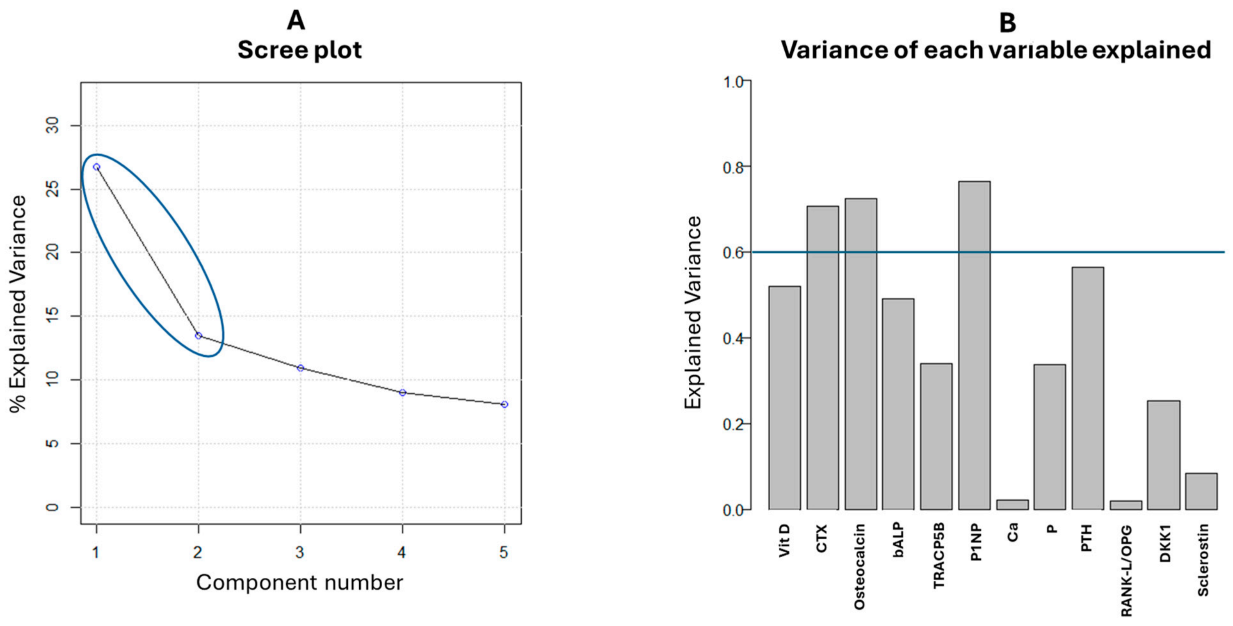

Figure S1. (A,B) Scree plot: % of explained variance vs. component number of principal component of the model (in general, the component number before the inflection point is retained). Variance of each variable explained: the weight of each variable to the two principal components selected in the PCA model.

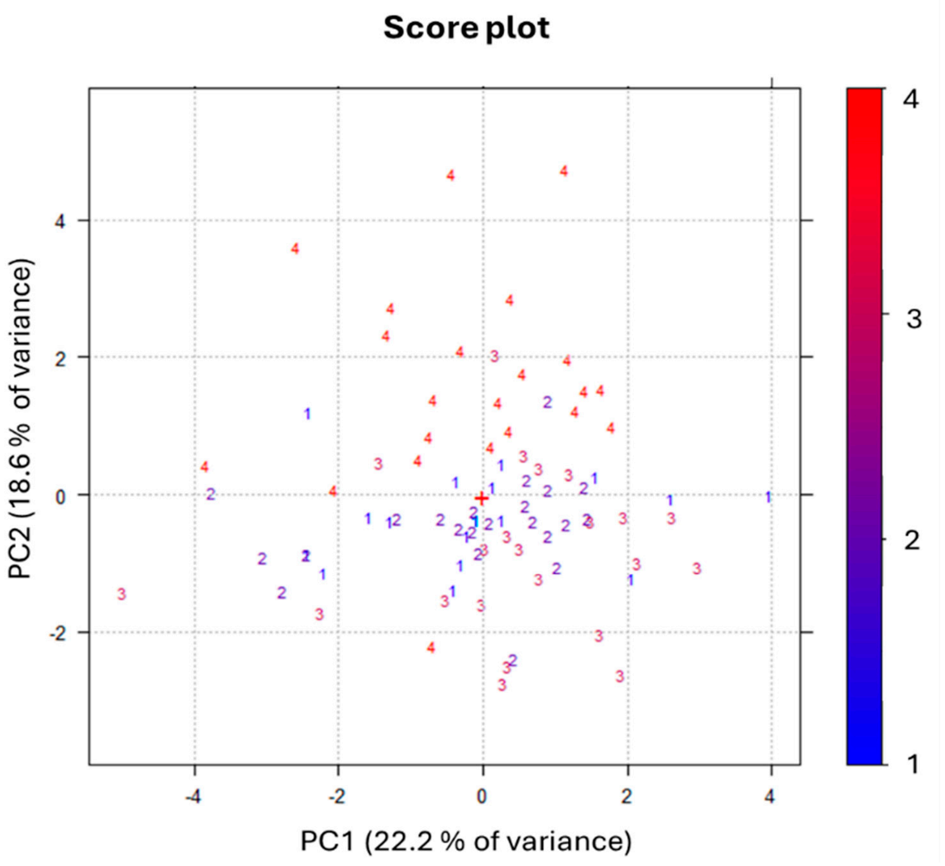

Figure S2. The score plot obtained considering data from all-time points, named from 1 to 4 with a color scale, except the onset time point.

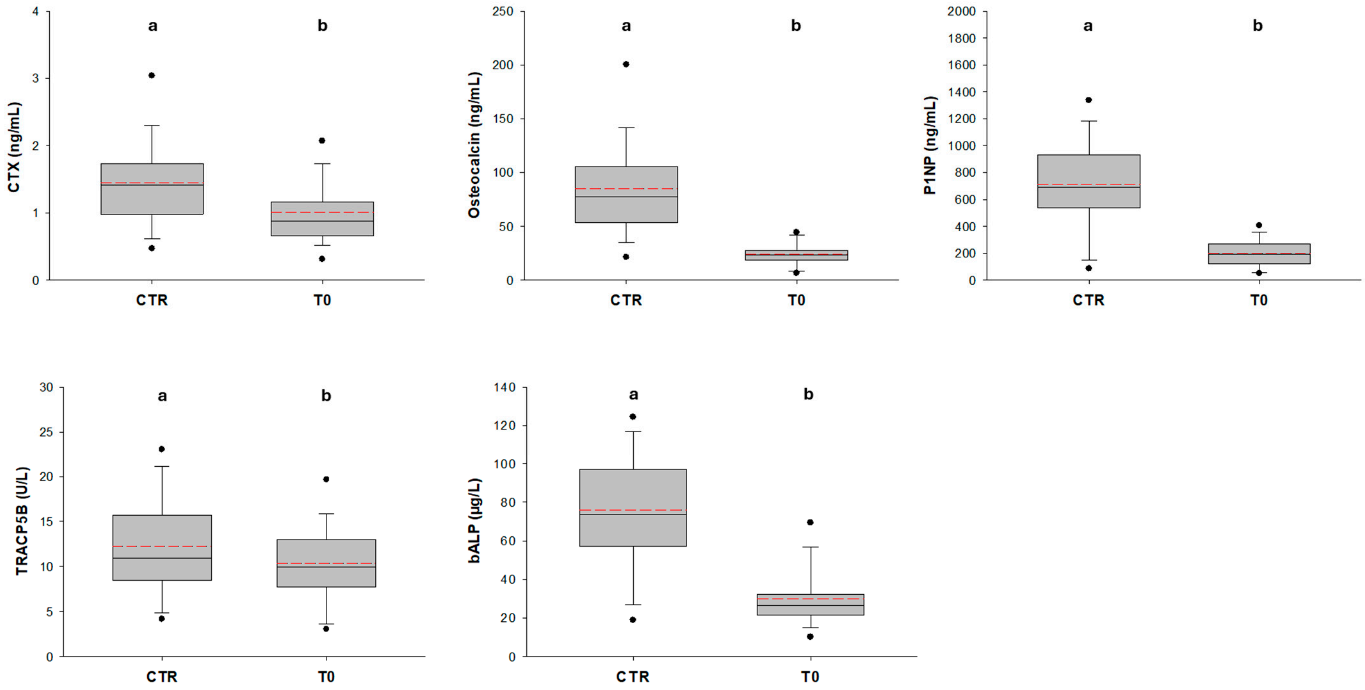

Figure S3. Distribution of bone remodeling markers, C-terminal-telopeptide-type-I-collagen (CTX), osteocalcin (OC), procollagen-type-I-N-terminal-propeptide (P1NP), bone-alkaline-phosphatase (bALP) and tartrate-resistant acid-phosphatase-5b (TRACP5b) measured for controls and patients at the onset time point. The mean and median values are marked by dotted red line and continuous black line, respectively. The different superscript letters in each box plot indicate statistical difference between the two groups ($p<0.05$).

Table S1. Bone metabolism and bone remodeling markers. Comparison between controls and patients at onset time point (T0).

Marker	CTR	T0	p-value
**CTX (ng/mL)**	1.4(1.0÷1.7)[a]	0.9(0.7÷1.2)[b]	**[1]0.009**
**Osteocalcin(ng/mL)**	77.7(53.3÷105.9)[a]	23.6(17.1÷28.2)[b]	**[1]<0.001**
**P1NP (ng/mL)**	692(531÷948)[a]	193(118÷273)[b]	**[1]<0.001**
**bALP (µg/L)**	71.7(56.0÷97.7)[a]	26.7(21.4÷32.3)[b]	**[1]<0.001**
**TRAcP5b (U/L)**	10.9(8.5÷16.4)[a]	10.0(7.6÷13.0)[a]	[1]0.308
**RANKL (pmol/L)**	1954(350÷7259)[a]	4302(1312÷20336)[b]	**[1]0.033**
**OPG (pmol/L)**	3.7(3.0÷4.5)[a]	4.4(2.9÷7.4)	**[1]0.030**
**RANKL/OPG**	581(89÷2346)[a]	920(311÷2838)[a]	[1]0.141
**DKK1 (pg/mL)**	5354±1342[a]	2921±1898[b]	**[2]<0.001**
**Sclerostin (pmol/L)**	17.8(15.3÷19.9)[a]	14.9(13.9÷18.3)[b]	**[1]0.023**

Data are presented as median with interquartile range as non-normally distributed variables. CTX: C-terminal telopeptide cross-links of type I collagen; bALP: total alkaline phosphatase; TRAcP5b: tartrate-resistant acid phosphatase; P1NP: procollagen type I N-terminal propeptide; DKK-1: Dickkopf-related protein 1; RANKL: soluble receptor activator of nuclear factor kappa-B ligand; OPG: osteoprotegerin. Different superscript letters above each value, in each line, indicate statistically significant differences between controls and patients at the onset time points. The significance level for all tests was set at 0.05.

[1]Nonparametric t-test (Mann-Whitney U test)
[2]Parametric t-test
